# Mathematical study of polycystic ovarian syndrome disease including medication treatment mechanism for infertility in women

**DOI:** 10.3934/publichealth.2024002

**Published:** 2023-12-04

**Authors:** Maryam Batool, Muhammad Farman, Aqeel Ahmad, Kottakkaran Sooppy Nisar

**Affiliations:** 1 Institute of Mathematics, Khwaja Fareed University of Engineering and Information Technology, Rahim Yar Khan, Pakistan; 2 Department of Computer Science and Mathematics, Lebanese American University, 1107-2020, Beirut, Lebanon; 3 Faculty of Arts and science, Mathematical research center, Near East University, Northern Cyprus, Turkey; 4 Department of Mathematics, Ghazi University, DG Khan, Pakistan; 5 Department of Mathematics, College of Science and Humanities in Alkharj, Prince Sattam Bin Abdulaziz University, Alkharj 11942, Saudi Arabia

**Keywords:** polycystic ovary syndrome, boundedness, uniqueness, reproductive number, stability

## Abstract

Among women of reproductive age, PCOS (polycystic ovarian syndrome) is one of the most prevalent endocrine illnesses. In addition to decreasing female fertility, this condition raises the risk of cardiovascular disease, diabetes, dyslipidemia, obesity, psychiatric disorders and other illnesses. In this paper, we constructed a fractional order model for polycystic ovarian syndrome by using a novel approach with the memory effect of a fractional operator. The study population was divided into four groups for this reason: Women who are at risk for infertility, PCOS sufferers, infertile women receiving therapy (gonadotropin and clomiphene citrate), and improved infertile women. We derived the basic reproductive number, and by utilizing the Jacobian matrix and the Routh-Hurwitz stability criterion, it can be shown that the free and endemic equilibrium points are both locally stable. Using a two-step Lagrange polynomial, solutions were generated in the generalized form of the power law kernel in order to explore the influence of the fractional operator with numerical simulations, which shows the impact of the sickness on women due to the effect of different parameters involved.

## 1. Introduction

One of the most prevalent endocrine system illnesses, polycystic ovarian syndrome (PCOS), affects 5 to 10% of women [1]. The PCOS is one of the most prevalent causes of ovulatory failure [2]. Women ages 18-44 are affected. PCOS develops during adolescence and is brought on by hormonal imbalances. Follicles, or cysts, which are fluid-filled sacs, are found on the ovary's periphery. A polycystic ovary (PCO) is defined as having twelve or more follicles that are between two to nine mm in diameter [3]. Both health and the standard of living for women are impacted by PCOS. Some of the symptoms include weight, worry, depression, and stress [4]. Others include heart issues, ovarian failure and infertility, type 2 diabetes, late menopause, acne, hair loss, baldness, and hirsutism [5]. According to reports, the prevalence ranges from 2.2–26% globally [6]. According to community research conducted in the United Kingdom (UK), South Asians exhibit a prevalence of 52% when compared to Caucasians, who exhibit a prevalence of 22% [3]. Early identification and treatment can be used to control the symptoms and avoid long-term problems. Gynaecologists urge patients on clomiphene citrate to wait no longer than six months after beginning their ovulation cycles before starting gonadotrophin therapy. The ovulation stimulation approach should not depend on whether the clomiphene citrate and gonadotropin therapy cycles are identified and their therapeutic follow-up is taken care of, but whether the pregnancy does not develop over the course of the 912 treatment cycles. Thus, it is encouraged to use assisted reproductive techniques (ART), among which in vitro fertilization (IVF) is one [7]. It has become increasingly obvious over the past ten years that PCOS patients need better clinical and therapeutic care. The primary attempts for better controlling PCOS were the adoption of more precise technologies for detecting circulating androgens, comprehension of the impact of PCOS on risk factors and ultimately, pharmaceutical regimens based on individual-specific phenotypic requirements [8]. Mathematical modeling can be used to suggest controls for any infectious disease after accounting for the disease's mechanism of transmission. Different nonlinear therapeutic rates between diseases are possible. The nonlinear incidence and treatment rate can be very helpful in assisting health organizations identify effective treatments that will stop the spread of the disease [9].

Various operators have been presented in fractional calculus. The Caputo operator, which is defined on a power law kernel, is the most fundamental operator. Many scientists working in engineering [10], mathematical biology [11], [12], fluid mechanics [13], [14], and mathematical physics [15]–[17] by using fractional derivative. A study on the numerical and computational aspects of the physical system of a (1+1)-dimensional Mikhailov-Novikov-Wang (MNW) integrable equation was conducted by Khater et al. [18]–[20]. The analytical and approximate solutions of the caudrrey-dodd-gibbon (CDG) model were examined by Khater et al. [21], while the computational simulations of the propagation of tsunami waves across the ocean were covered in [22]. Particularly, fractional calculus has also been used to study the dynamics of cancer [23]. A fractional component cancer model was put forth by Naik et al. [24]. Atangana [25] put out a novel family of two-parameter derivatives in 2017. The order of the two parameters, where one reflects the exponential decay, power law, or Mittag-Leffler kernel operations, describes the fractal dimension. It is impossible to obtain certain nonlinear model parameters through experimentation. This work suggests fractal-fractional derivatives as a potential solution to these problems. Farman et al. proposed in [26] a Caputo Fabrizio fractional order model for glucose control in insulin therapy for diabetes. Researchers expanded hybrid fractal-fractional operators and discussed a whole new hybrid model of the coronavirus propagation in [27]. They also compared its results with past iterations of the fractal-fractional model. To understand how the Ebola virus spreads, Farman et al. [28] proposed a nonlinear time-fractional mathematical model of the disease. The fractional descement's stripping endothelial keratoplasty (DSEK) model was examined using fractional derivatives of the Atangana-Baleanu Caputo type [29]. The fractional Euler's numerical approach was derived using the Atangana-Baleanu Caputo (ABC) fractional derivative and was then applied to the fractional DSEK model. Researchers used fractal-fractional Atangana-Baleanu derivatives and integrals in the sense of Caputo to study the dynamics of Q (query) fever transmission in cattle and ticks as well as the bacterial load in the environment [30]. The freshly created Newton polynomial was utilized by the stated numerical approach. Researchers researched and observed the dynamical transmission of the illness under the impact of vaccination using a unique fractional order measles model, utilizing a constant proportional (CP) Caputo operator [31]. To express a set of fractional differential equations numerically, they used Laplace with the Adomian decomposition approach. A generalized fractional model was used to describe how HIV/AIDS spread throughout the Cape Verde Islands [32]. The model was successfully built using a two-step Lagrange polynomial interpolation, and the associated error analysis was investigated. In [33], a fractional order tubarculosis (TB) model with a fractal fractional operator was built using an expanded Mittag-Leffler kernel.

This is how the sections are organized: Literature evaluation and an introduction are provided in section one. Basic terms related to fractional derivatives are covered in section two. We spoke about the fractional-order PCOS model with the Mittag-Leffler kernal in section three. In section 3.1, we prove the detailed analysis of positiveness and boundedness, existence and uniqueness, reproductive number, and equilibrium point analysis. In section four, the equilibrium point stability analysis with Jacobian matrix and Lyaounov function is performed. In section five, numerical results are developed.

## 2. Basic concepts

### 2.1. Definition 1 [34]

Consider x∈k(c,d),d>c,η∈[0,1], for the Caputo derivative of arbitrary is given by

$$D_{t}^{\alpha }x(t)=\frac{L(\alpha )}{1-\alpha }\int_{c}^{t}x^{\prime }(t)exp[-\alpha \frac{t-\varsigma }{1-\alpha }]d\varsigma ,$$ (1)

where *L*(*α*) is the normalization of the function that holds *L*(0) = *L*(1) = 1.

However, if j∉k(c,d), we get

$$D_{t}^{\alpha }x(t)=\alpha \frac{L(\alpha )}{1-\alpha }\int_{c}^{t}(x(t)-x(\varsigma ))exp[-\alpha \frac{t-\varsigma }{1-\alpha }]d\varsigma .$$ (2)

Remark: If $\kappa =\frac{1-\alpha }{\alpha }\in [0,1]$, then we get the form

$$D_{t}^{\alpha }h(t)=\frac{R(\kappa )}{\kappa }\int_{c}^{t}x^{\prime }(t)exp[-\frac{t-\varsigma }{\kappa }]d\varsigma ,$$ (3)

G(0)=G(∞)=1. Moreover ${lim}_{\kappa \to \infty }\frac{1}{\kappa }exp[-\frac{t-\varsigma }{\kappa }]=\lambda (\varsigma -t).$

### 2.2. Definition 2 [34]

The Caputo fractional derivative is given by *c* > 0, $q\in H_{1}(0,c)$ and 0<η<1; thus

(4) \begin{equation*} ^CD^{\alpha} x(t) = \frac{1}{\Gamma(1-\alpha)} \int_0^t (t-\varsigma)^{-\alpha} (x^\prime (\varsigma) d\varsigma. t>0 \end{equation*}

### 2.3. Definition 3 [35], [36]

A power law kernel in the Riemann-Liouville concept is defined as;

(5) \begin{eqnarray*} _0^{FFP}D_t^{\alpha,\upsilon} x(t) = \frac{1}{\Gamma (1- \alpha)} \frac{d}{dt^\upsilon} \int_0^t (t-\varsigma)^{- \alpha} x(\varsigma) d\varsigma, \end{eqnarray*}

with 0≤α,υ≤1,

$$\frac{df(t)}{dt^{\upsilon }}=\underset{t\to t_{1}}{lim}\frac{f(t)-f(t_{1})}{t^{2-\upsilon }-t_{1}^{2-\upsilon }}(2-\upsilon ).$$

The fractal-fractional integral corresponding power law kernel of order (α,υ) is given as

(6) \begin{eqnarray*} _0^{FFP}I_t^{\alpha,\upsilon} x(t) = \frac{1}{\Gamma(\alpha)}\int_0^t (t - \varsigma)^{\alpha - 1} \varsigma^{1- \upsilon} x(\varsigma) d\varsigma. \end{eqnarray*}

## 3. Polycystic ovarian syndrome model with fractional derivative

Considering the classical order model given in [7], in our assumption, put second treatment factor zero for better understanding transmission of disease in society in different age groups. The model is predicated on the following hypotheses: the whole population, or *N*(*t*), is composed of five sub-populations: Women who are *S*(*t*) susceptible to infertility, *I*(*t*) suffering from PCOS, *T*(*t*) infertile women receiving treatment with gonadotropin and clomiphene citrate and *R*(*t*) women who have recovered from infertility. The following nonlinear fractional differential equations for the fractional order model are created using the fractal-fractional operator in the Caputo sense:

(7) \begin{eqnarray*} _0^{FFP}D_t^{\alpha,\upsilon} S(t) &=& \Pi - a S - b e S T \nonumber\\ _0^{FFP}D_t^{\alpha,\upsilon} I(t) &=& b e S T - (a + k)I \nonumber\\ _0^{FFP}D_t^{\alpha,\upsilon} T(t) &=& k c I - (r+a)T \\ _0^{FFP}D_t^{\alpha,\upsilon} R(t) &=& u r T - a R, \nonumber \end{eqnarray*}

with initial state $S(0)=S_{0}\geq 0,I(0)=I_{0}\geq 0,T(0)=T_{0}\geq 0,R(0)=R_{0}\geq 0$,

where *a* represents the frequency at which patients visit the clinic for a disease diagnosis and treatment. Abortion and restarting the treatment cycle occur at a rate of *b*. The rate of therapy for women who got pregnant with medicine (gonadotropin and clomiphene citrate) is *e*. We display the treatment rate in the patient class with *k* as well as the *k* patient group who were receiving *c* medical therapy. We use *r* to display the group *T*'s recovery rate. We represent the number of recoveries as *urT*, where *u* is the recovery rate of *rT* at time *t*. There were *aR* susceptible individuals who died and left the group. The number of infertile women who used a therapeutic therapy is represented by Π=jN.

### 3.1. Analysis of proposed model

#### 3.1.1. Solution boundedness and positivity

To demonstrate the positivity of the solutions since they reflect actual problems in the real world with positive values, in this subsection, we look at the circumstances in which the considered model's solutions satisfy the positivity requirement.

**Theorem 3.1** S(t),I(t),T(t) and *R*(*t*) are positive solutions to the system equation for all t≥0 if S(0)≥0,I(0)≥0,T(0)≥0,R(0)≥0, respectively. All of the solutions to the system of equations S(t),I(t),T(t) and *R*(*t*) are finite.

Let's begin with the *S*(*t*) group:

$$\begin{array}{l} S(t)=\Pi -aS-beST,\forall t\geq 0\\ S(t)\geq \Pi -aS-beST,\\ S(t)\geq -(a+beT)S. \end{array}$$ (8)

The norm defined as

$${\left\|h\right\|}_{\infty }=sup_{t\in D_{h}},$$ (9)

$$\begin{array}{l} S(t)\geq -(be|T|+a)S\\ S(t)\geq -(abe{\left\|T\right\|}_{\infty }+a)S. \end{array}$$ (10)

This yields

$$S(t)\geq S^{0}e^{-(a+be{\left\|T\right\|}_{\infty })},$$ (11)

$$\begin{array}{l} I(t)=beST-(k+a)I,\forall t\geq 0\\ \geq beST-(a+k)I\\ \geq -(k+a)I, \end{array}$$ (12)

which then yields

$$I(t)\geq I^{0}e^{-(a+k)}.$$ (13)

Repeating the process for other classes finds the following inequalities:

$$T(t)\geq T^{0}e^{-(r+a)}$$ (14)

$$R(t)\geq R^{0}e^{-a}.$$ (15)

### 3.2. Positive solutions with nonlocal operator

All system (7) findings are favorable if the beginning conditions for nonlocal operators are satisfied.

With regard to the power law kernel and the fractal-fractional operator, we obtain

$$\begin{array}{l} S(t)\geq E_{\alpha }S(0)(-q^{1-\upsilon }(a+be{\left\|T\right\|}_{\infty })t^{\alpha }),\forall t\geq 0\\ I(t)\geq E_{\alpha }I(0)(-q^{1-\upsilon }(a+k)t^{\alpha }),\forall t\geq 0\\ T(t)\geq E_{\alpha }T(0)(-q^{1-\upsilon }(a+r)t^{\alpha }),\forall t\geq 0\\ R(t)\geq E_{\alpha }R(0)(-q^{1-\upsilon }(a)t^{\alpha }),\forall t\geq 0. \end{array}$$ (16)

where *q* is time component.

### 3.3. Positively invariant region

**Theorem 3.1** *The PCOS model's suggested solution is separate and limited in $R_{+}^{4}$ under straight-line circumstances*.

**Proof.** We will look at the positive solution of the system (7), which is as follows:

17 \begin{eqnarray*} _0^{FFP}D_t^{\alpha,\upsilon} S(t)|_{S=0} &=& \Pi \geq 0,\nonumber\\ _0^{FFP}D_t^{\alpha,\upsilon} I(t)|_{I=0} &=& b e S T \geq 0,\nonumber\\ _0^{FFP}D_t^{\alpha,\upsilon} T(t)|_{T=0} &=& k c I \geq 0 ,\\ _0^{FFP}D_t^{\alpha,\upsilon} R(t)|_{R=0} &=& u r T \geq 0.\nonumber \end{eqnarray*}

The solution is unable to escape the hyperplane if $(S(0),I(0),T(0),R(0))\in R_{+}^{4}$. The vector field on each hyperplane around the nonnegative orthant also points into the domain $R_{+}^{4}$, making it a positivity invariant set.

### 3.4. Existence and uniqueness analysis

In this part, we used the fixed-point theory to discuss the presence and distinctiveness of the proposed system. Schauder's fixed-point theorem guarantees the model's existence, while Banach's contraction theorem guarantees its singularity. By applying a fractional derivative in the Caputo sense for 0<α≤1, system (7) can be generalized as illustrated below:

(18) \begin{eqnarray*} _0^{FFP}D_t^{\alpha,\upsilon} S(t) &=& \Pi - a S - b e S T \nonumber\\ _0^{FFP}D_t^{\alpha,\upsilon} I(t) &=& b e S T - (a + k)I \nonumber\\ _0^{FFP}D_t^{\alpha,\upsilon} T(t) &=& k c I - (a + r)T \\ _0^{FFP}D_t^{\alpha,\upsilon} R(t) &=& u r T - a R, \nonumber \end{eqnarray*}

with initial state $S(0)=S_{0},I(0)=I_{0},T(0)=T_{0},R(0)=R_{0}$. Let

$$\begin{array}{l} \Delta_{1}(S,I,T,R,t)=\Pi -aS-beST\\ \Delta_{2}(S,I,T,R,t)=beST-(a+k)I\\ \Delta_{3}(S,I,T,R,t)=kcI-(a+r)T\\ \Delta_{4}(S,I,T,R,t)=urT-aR. \end{array}$$ (19)

Using initial condition and fractional integral, we have

$$S(t)=S_{0}+\frac{1}{\Gamma (\alpha )}\int_{0}^{t}{\left(t-\zeta \right)}^{\alpha -1}\Delta_{1}(S,I,T,R,t)d\zeta ,$$ (20)

$$I(t)=I_{0}+\frac{1}{\Gamma (\alpha )}\int_{0}^{t}{\left(t-\zeta \right)}^{\alpha -1}\Delta_{2}(S,I,T,R,t)d\zeta ,$$ (21)

$$T(t)=T_{0}+\frac{1}{\Gamma (\alpha )}\int_{0}^{t}{\left(t-\zeta \right)}^{\alpha -1}\Delta_{3}(S,I,T,R,t)d\zeta ,$$ (22)

$$R(t)=R_{0}+\frac{1}{\Gamma (\alpha )}\int_{0}^{t}{\left(t-\zeta \right)}^{\alpha -1}\Delta_{4}(S,I,T,R,t)d\zeta .$$ (23)

Let

$$\Lambda (t)=(\begin{array}{c} S(t)\\ I(t)\\ T(t)\\ R(t) \end{array},\Lambda_{0}=(\begin{array}{c} S_{0}\\ I_{0}\\ T_{0}\\ R_{0} \end{array},ℵ(\zeta ,\Lambda (\zeta )=(\begin{array}{c} \Delta_{1}(S,I,T,R,t)\\ \Delta_{2}(S,I,T,R,t)\\ \Delta_{3}(S,I,T,R,t)\\ \Delta_{4}(S,I,T,R,t). \end{array}$$ (24)

Thus, system (20) becomes

$$\Lambda (t)=\Lambda_{0}+\frac{1}{\Gamma (\alpha )}\int_{0}^{t}ℵ(\zeta ,\Lambda (\zeta )(t-\zeta {)}^{\alpha -1}d\zeta .$$ (25)

Now, consider a Banach space ℘[0,T]=ϖ with a norm

$$\left\|S,I,T,R\right\|=\underset{t\in [0,T]}{max}[|S,I,T,R|].$$ (26)

Let a mapping be defined as $\diamondsuit:\varpi \rightarrow \varpi$, then

27 \begin{equation*} \diamondsuit \Lambda(t)=\Lambda_0 + \frac{1}{\Gamma(\alpha)} \int_0^t (t-\zeta)^{\alpha - 1} \aleph(\zeta , \Lambda(\zeta) d\zeta. \end{equation*}

In addition, we impose the following hypothesis on a nonlinear function:

P1) There exist constants $\rho_{m},\rho_{m}>0$, such that

$$|ℵ(t,\Lambda (t)|\leq \rho_{m}|\Lambda (t)|+\rho_{m}.$$ (28)

P2) There exists a constant $L_{m}>0$ for each $\Lambda ,\bar{\Lambda }\in \varpi$ such that

$$|ℵ(t,\bar{\Lambda })-ℵ(t,{\bar{\Lambda }}_{1})|\leq L_{m}|\bar{\Lambda }-{\bar{\Lambda }}_{1}|.$$ (29)

**Theorem 3.2** *The system (18) has at least one solution if the assumptions* (*P*1) *are true*.

**Proof.** To demonstrate that $\diamondsuit$ is bounded, let Φ={Λ∈Φ|ρ≥‖Λ‖}, where

$$\rho \geq \underset{t\in [0,T]}{max}\frac{\Lambda_{0}+\left(\frac{\rho_{m}T^{\alpha }}{\Gamma (\alpha +1)}\right)}{1-\left(\frac{\rho_{m}T^{\alpha }}{\Gamma (\alpha +1)}\right)}$$ (30)

is a closed convex subset of ϖ. Now,

31 \begin{eqnarray*} \Vert \diamondsuit \Lambda \Vert &=& \max_{t \in [0,\mathbf{T}]}\vert \Lambda_0 + \frac{1}{\Gamma(\alpha)} \int_0^t (t-\zeta)^{\alpha - 1} \aleph(\zeta , \Lambda(\zeta) d\zeta \vert \nonumber\\ &\leq & \vert \Lambda_0 \vert + \frac{1}{\Gamma(\alpha)} \int_0^t (t-\zeta)^{\alpha - 1} \vert \aleph(\zeta , \Lambda(\zeta)\vert d\zeta \nonumber\\ &\leq & \vert \Lambda_0 \vert + \frac{1}{\Gamma(\alpha)} \int_0^t (t-\zeta)^{\alpha - 1} [\rho_m \vert \Lambda (t) \vert + \varrho_m ] d\zeta \\ &\leq & \vert \Lambda_0 \vert + [\rho_m \Vert \Lambda \Vert + \varrho_m ] \frac{\mathbf{T}^{\alpha}}{\Gamma(\alpha + 1)} \leq \rho.\nonumber \end{eqnarray*}

Since $ \Lambda \in \Phi \Rightarrow \diamondsuit (\Phi) \subseteq \Phi$, it shows that $\diamondsuit$ is bounded. Let $t_{1}<t_{2}\in [0,T]$ to prove that $\diamondsuit$ is completely continuous and take

32 \begin{eqnarray*} \Vert \diamondsuit \Lambda(t_2) - \diamondsuit \Lambda(t_1) \Vert &=& \vert \frac{1}{\Gamma(\alpha)} \int_0^{t_2} (t_2-\zeta)^{\alpha - 1} \aleph(\zeta , \Lambda(\zeta) d\zeta \nonumber\\ & - & \frac{1}{\Gamma(\alpha)} \int_0^{t_1} (t_1-\zeta)^{\alpha - 1} \aleph(\zeta , \Lambda(\zeta) d\zeta \vert \\ &\leq & [\rho_m \Vert \Lambda \Vert + \varrho_m ] \frac{[t_2^{\alpha} - t_1^{\alpha}]}{\Gamma(\alpha + 1)}.\nonumber \end{eqnarray*}

This shows that $\Vert \diamondsuit \Lambda(t_2) - \diamondsuit \Lambda(t_1) \Vert \rightarrow 0$ as $t_{2}\to t_{1}$. The Arzela-Ascoli theorem proves that the operator $\diamondsuit$ is entirely continuous. According to Schauder's fixed point theorem, there is at least one solution for the given system (18). The system (18) was subsequently shown to have an exclusive solution using the Banach fixed point theorem.

**Theorem 3.3** *Suppose that if the requirements* (*P*2) *are met, system (18) has a unique (one) solution*.

**Proof.** Let $\bar{\Lambda}, \bar{\Lambda}_1 \in \varpi$ and take

33 \begin{eqnarray*} \Vert \diamondsuit (\bar{\Lambda}) - \diamondsuit (\bar{\Lambda}_1) \Vert &=& \max_{t \in [0,\mathbf{T}]}  \vert \frac{1}{\Gamma(\alpha)} \int_0^{t} (t_2-\zeta)^{\alpha - 1} \aleph(\zeta , \bar{\Lambda}(\zeta) d\zeta \nonumber\\  &-& \frac{1}{\Gamma(\alpha)} \int_0^{t} (t_1-\zeta)^{\alpha - 1} \aleph(\zeta , \bar{\Lambda}_1(\zeta) d\zeta \vert \\ &\leq & \frac{T^{\alpha}}{\Gamma(\alpha + 1)} L_m \vert \bar{\Lambda} - \bar{\Lambda}_1 \vert.\nonumber \end{eqnarray*}

Hence, the $\diamondsuit$ is the contraction. By the Banach fixed point (BFP) theorem, system (18) has a unique solution.

### 3.5. Equilibrium points analysis

This section provides a comprehensive analysis of equilibrium points. For equilibrium points, we solve the system shown below:

$$\begin{array}{l} \Pi -aS^{*}-beS^{*}T^{*}=0\\ beS^{*}T^{*}-(a+k)I^{*}=0\\ kcI^{*}-(a+r)T^{*}=0\\ urT^{*}-aR^{*}=0. \end{array}$$ (34)

The disease free points are

$$E^{0}\left(\frac{\Pi }{a},0,0,0\right).$$

Thus, if $I^{*}=0$, then we have $S^{*}=\frac{\Pi }{a},T^{*}=0,R^{*}=0$, which are free disease equilibrium points. The endemic equilibrium points at this time are

$$E^{*}\left(S^{*},I^{*},T^{*},R^{*}\right),$$

$$E^{*}\left(\frac{\left(a+k)(a+r\right)}{bkec},\frac{\Pi bkec-a(a+k)(a+r)}{bkec(a+k)},\frac{\Pi bkec-a(a+k)(r+a)}{be(a+k)(r+a)},\frac{ur(\Pi bkec-a(k+a)(a+r))}{abe(a+k)(a+r)}\right).$$

### 3.6. Reproduction number

Consider the following equation to get the reproduction number:

(35) \begin{eqnarray*} _0^{FFP}D_t^{\alpha,\upsilon} I(t) &=&  b e S T - (a + k)I \nonumber\\ _0^{FFP}D_t^{\alpha,\upsilon} T(t) &=& k c I - (a + r)T. \end{eqnarray*}

The next generation matrix technique must now be used to compute the matrices *F* and *V*^−1^ as follows:

$$F=\left[\begin{array}{cc} 0 & \frac{\Pi be}{a}\\ 0 & 0 \end{array}\right],V^{-1}=\left[\begin{array}{cc} \frac{1}{\left(a+k\right)} & 0\\ \frac{kc}{\left(a+k)(a+r\right)} & \frac{1}{\left(a+r\right)} \end{array}\right],$$ (36)

then the reproduction number is obtained as

$$R_{0}=\frac{\Pi bkec}{a(a+k)(a+r)}.$$ (37)

**Figure 1.**
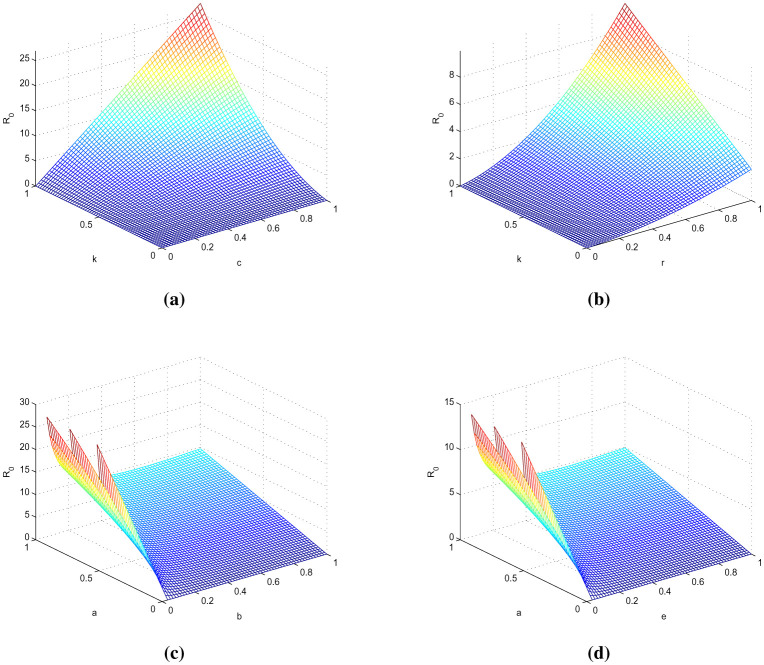
Analysis of different parameters.

## 4. Stability analysis

### 4.1. Local stability of the disease-free equilibrium point

**Theorem 4.1** *If the effective reproduction number is R*_0_ *<* 1*, at equilibrium point $E^{0}\left(\frac{\Pi }{a},0,0,0\right)$ of model (7) is locally asymptotically stable, and if R*_0_ > 1*, it is unstable*.

**Proof.** The Jacobian matrix *J*(*E*^0^) of model (7) with respect to (*S*, *I*, *T*, *R*) at the without equilibrium point $E^{0}\left(\frac{\Pi }{a},0,0,0\right)$ is as follows:

$$J(E^{0})=\left[\begin{array}{cccc} -a & 0 & -\frac{\Pi be}{a} & 0\\ 0 & -(a+k) & \frac{\Pi be}{a} & 0\\ 0 & kc & -(a+r) & 0\\ 0 & 0 & ur & -a \end{array}\right].$$

When it happens, the associated characteristic equation is

$$(-a-\Upsilon_{1})(-(a+k)-\Upsilon_{2})(-(a+r)-\Upsilon_{3})(-a-\Upsilon_{4})=0,$$ (38)

$\Upsilon_{1}=-a$ or $\Upsilon_{2}=-(a+k)$ or $\Upsilon_{3}=-(a+r)$ or $\Upsilon_{4}=-a.$

As a result, every root is negative. Hence, the disease-free equilibrium point is unstable if the effective reproduction number is *R*_0_ < 1 and is locally asymptotically unstable otherwise.

**Table 1.** Comparative analysis.

**Year**	**Authors**	**Contribution**
2023	Alamoudi et al. [2]	In this paper, a data set containing an ultrasound image of the ovary and clinical information about a patient classified as either PCOS or non-PCOS is presented.
2023	A. Chaudhuri [5]	Women are frequently advised by society to conceal physical issues like PCOS. This study covers the aetiology, diagnosis, causes, symptoms, and potential treatments for PCOS, including medication, herbal remedies, acupuncture, and bariatric surgery.
2022	A. S. Chauhan [4]	Obesity, inactivity, and a family history of PCOS are risk factors for PCOS. Treatment for PCOS may include changes to one's lifestyle, such as frequent exercise and weight loss. Many of the consequences can be prevented with early identification and treatment.
2019	S. Hafezi et al. [7]	The authors of this paper provide a dynamic model designed to forecast the outcome of treatment for infertile women who have polycystic ovarian syndrome.

## 5. Numerical simulation

**Figure 2.**
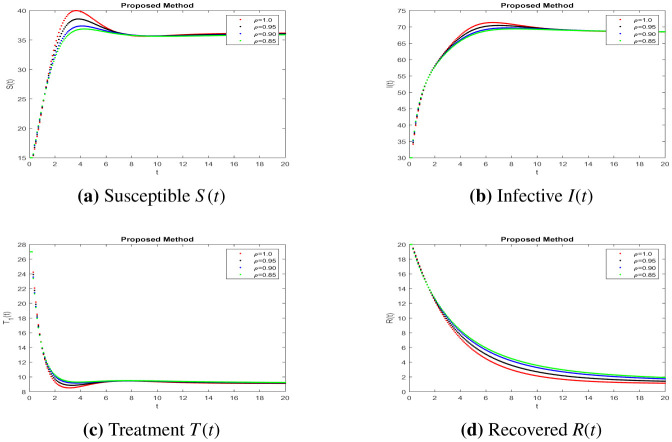
Simulation of the PCOS model compartments at different parameter values *k* = 0.47, *a* = 0.3, *j* = 0.5.

**Figure 3.**
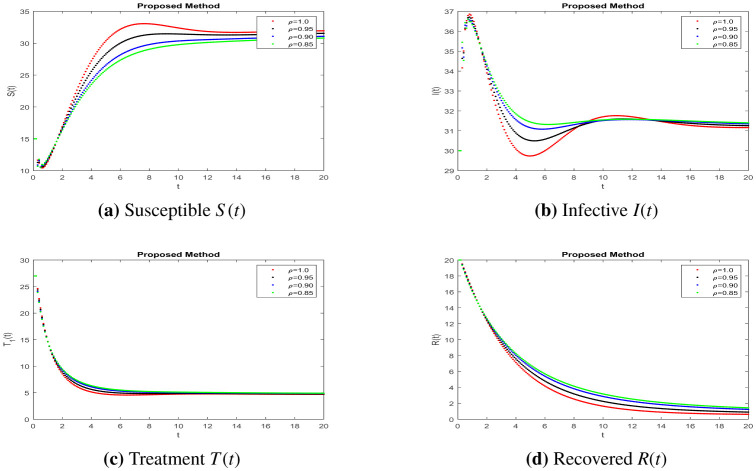
Simulation of the PCOS model compartments at different parameter values *k* = 0.47, *a* = 0.16, *j* = 0.23.

In this part, solutions are obtained using the MATLAB software and the fractal fractional (FF) operator in the Caputo sense. The proposed system's parameter values with initial conditions are as follows: b=0.24,e=0.47,c=0.34,r=0.9,u=0.04 and $S_{0}=15,I_{0}=30,T_{0}=27,R_{0}=20,N_{0}=104$, as given in [7]. To assess the model's qualitative behavior, the influence of its system characteristics and potential controls with different values of parameters are shown in Figures 1 to 3 at different fractional order values. Simulation of the system at k=0.47,a=0.3 and j=0.5 is in Figure 1 of different compartments. Here, we can observe better results when shot for a short time and no response in treatment at higher ages given in [7], having a better understanding of the impact of medication on patients. The simulation of the system at k=0.47,a=0.16,j=0.23 and k=0.1,a=0.16,j=0.23, respectively, is covered in Figures 2-3 and shows the impact of treatment, where we can observe the susceptible population increase and the infection rate decrease when treatment continues with time. In recent decades, medical therapy for women's difficulties has included the use of gonadotropin and clomiphene citrate. Gonadotrophin or clomiphene citrate are two oral medications that can be used to trigger ovulation in anovulatory PCOS individuals who wish to get pregnant. Drugs called gonadotropin enhance the release of gonadotrophin hormones and cause ovulation, while clomiphene lowers estrogen levels and raises gonadotrophin-releasing hormone (GnRH) levels. With different values of influencing parameters in several age groups, the impact of treating compartments is shown in Figures 2(a-d). Figures 3(a-d) shows better fixing of fractional and fractal parameters, while infection compartments in Figure 3(b) bounded to zero show an improved recovered population at fractional value. The persistence of the disease in the population affects how mathematical models are analyzed. This model assumes that the population volume is stable throughout time and offers a reliable estimate for brief periods with similar patient conditions in early ages. Here, we can also observe the memory effect of fractal and fractional order derivatives in simulations as compared to ordinary derivatives in this model.

**Figure 4.**
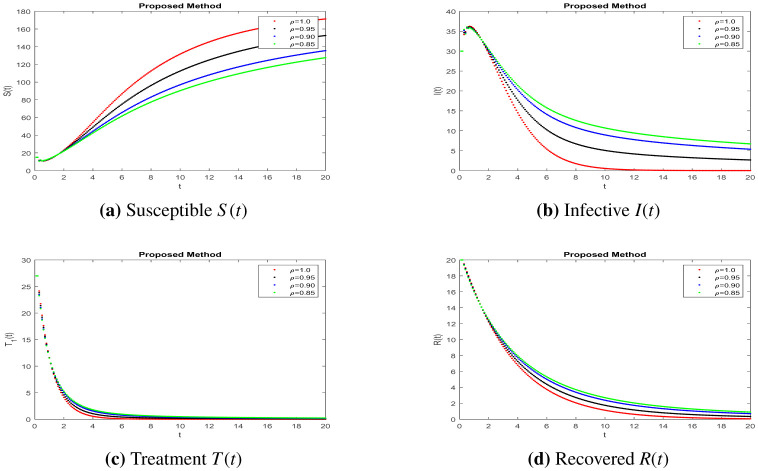
Simulation of the PCOS model compartments at different parameter values *k* = 0.1, *a* = 0.16, *j* = 0.23.

## 6. Conclusion

PCOS patients should first follow a healthy eating regimen. Clinicians should create customized dietary formulas, which include macronutrient ratios, micronutrient intake, and total calorie limitation, based on a comprehensive review of the patient's prior dietary composition. Second, exercise helps patients lose weight and also modifies their body composition. PCOS patients require a customized workout regimen that considers both muscle gain and fat loss, depending on their specific muscle fat ratios. In this article we proposed a fractional order model to predict the outcomes of treatment for infertile women with PCOS as a contributing factor through medication. Using the power law kernel, we developed a fractal-fractional model for observing the treatment impact and transmission of disease in society. We verified the important properties of the epidemic models, such as their positivity, boundedness, positive invariant region, equilibrium points, existence, and uniqueness of their solutions. The analysis of the local stability was done using the Jacobian matrix approach. The examination of the Lyapunov function for global stability was supported by the first and derivative tests. To analyze the effects of the fractional operator with numerical simulations, two-step Lagrange polynomial solutions were constructed. This demonstrates the impact of the sickness on women due to the effect of the many factors involved. According to numerical simulations that support analytical solutions, the dynamics of the development of PCOS are influenced by the fractal-fractional derivatives and they can reduce the spread of the condition in the population. Infertility is a frequent concern for PCOS women. Women with PCOS should be inspired by the possibility of a healthy pregnancy and successful conception. For PCOS-afflicted women should get the right advice and support while seeking medical attention. In this work, we contain a model by using Caputo operator lies properties of kernel which is local and non-singular. This can not implement for a nonlocal operator. In future work we proposed a new fractional model by using nonlocal kernel and optimal control stability and treatment plans that will also include other factors, including food, exercise and everyday activities.

## Use of AI tools declaration

The authors declare they have not used Artificial Intelligence (AI) tools in the creation of this article.
